# Supplementary material for: Association between ischemic stroke and seropositive rheumatoid arthritis in Korea: A nationwide longitudinal cohort study
Source: PLoS One. 2021 May 17;16(5):e0251851. doi: 10.1371/journal.pone.0251851 (PMC8128246; doi:10.1371/journal.pone.0251851)
Supplement: Attachment — Submitted filename: Response to Reviewers.docx [file pone.0251851.s001.docx]

**Title of the Manuscript**:

**Association between ischemic stroke and seropositive rheumatoid arthritis in Korea: A nationwide longitudinal cohort study**

**Manuscript Number: PONE-D-20-30296**

***Editor***

**METHODS:**

**Comment a)**

**a) Clear inclusion and exclusion criteria should be written to understand both populations.**

**Author’s Response:**

I appreciate your valuable comment and I understand your concern. The NHIS-HEALS cohort included 514,557 participants, approximately 10% of the Korean population from 2002 to 2003, aged between 40 and 79 and who had undergone national health screening. It was followed up for 12 years, until December of 2015. The study population included a seropositive RA group and a control group. The seropositive RA group consisted of subjects with a diagnosis of seropositive RA (any ICD-10 code starting with M05) with a prescription of any disease-modifying anti-rheumatic drug (DMARD) between January 1, 2004, and December 31, 2015. We extracted 9,120 seropositive RA subjects from a total of 514,557 patients in the NHIS-HEALS cohort. After removing 1,384 subjects with preexisting seropositive RA, a total of 7,736 patients with newly diagnosed seropositive RA remained. After exclusion of those without a prescription for any DMARD, 2765 patients with RA were retained. After applying this inclusion criteria, 2765 patients with newly diagnosed RA were established between 2004 and 2015 as our base cohort from the NHIS-HEALS cohort for analysis. Through 1:5 age- and sex-stratified matching (without replacement) using a greedy-match algorithm of the R package “MatchIT,” 13,825 individuals were chosen as controls (Fig. 1). Subjects in this study were followed up from the first occurrence of ischemic stroke until death or end of follow-up. In response to your comments, we have described the criteria clearly as follows.

**Page 4, line 92**

The NHIS-HEALS cohort included 514,557 participants, approximately 10% of the Korean population from 2002 to 2003, aged between 40 and 79 and who had undergone national health screening. It was followed up for 12 years, until December of 2015 [1]. Rheumatoid factor (RF) and anti-cyclic citrulline peptide (CCP) antibody tests are often measured to diagnose rheumatoid arthritis (RA); however, when we consider the tests may not be repeated, it is difficult to extract correctly from electronic health record data. Considering these points, we used M5(seropositive) diagnostic ICD-10 code based on these factors [2]. The study population included a seropositive RA group and a control group. The seropositive RA group consisted of subjects with a diagnosis of seropositive RA (any ICD-10 code starting with M05) with a prescription of any disease-modifying anti-rheumatic drug (DMARD) between January 1, 2004, and December 31, 2015 [25,27]. Our study based on people who received conventional synthetic DMARD prescriptions such as hydroxychloroquine, methotrexate, sulfasalazine and leflunomide. Biological DMARD medications were examined whether adalimumab, etanercept, infliximab, or rituximab were prescribed. We extracted 9,120 seropositive RA subjects from a total of 514,557 patients in the NHIS-HEALS cohort. After excluding 1,384 subjects with preexisting seropositive RA, a total of 7,736 patients with newly diagnosed seropositive RA remained. After exclusion of those without a prescription for any DMARD, 2765 patients with RA were retained. After applying this selection criteria, 2765 patients with newly diagnosed RA were identified between 2004 and 2015 as our base cohort from the NHIS-HEALS cohort for analysis. Through 1:5 age- and sex-stratified matching (without replacement) using a greedy-match algorithm of the R package “MatchIT” [29,30], 13,825 individuals were chosen as controls (Fig. 1). Subjects in this study were followed up from the first occurrence of ischemic stroke until death or end of follow-up.

**Comment b)**

**b) In terms of specific stroke risk, basic data from this condition should be provided, to understand if certain covariates could affect mortality and functional outcomes: severity of stroke cases (according to NIHSS),** **etiology of ischemic stroke (according to ASCOD,** **TOAST or any other classification criteria), type of acute therapy (IV thrombolysis, thrombectomy, both, or usual medical care), admission to stroke unit, general or rheumatology ward. Please clarifiy if this data is available, as I understand you adquire data from the NHID.**

**Author’s Response:**

I appreciate your valuable comment and I understand your concern. In this study, the material was obtained from a database by the NHIS-HEALS cohort. The NHIS-HEALS cohort covers approximately 10% of the Korean population from 2002 to 2003, aged between 40 and 79 and who had undergone national health screening. Because only deidentified data can be provided, we could not obtain all of each individual’s clinical information from the NHIS-HEALS cohort. It was impossible to know the mortality and functional outcomes according to the severity of stroke cases. Although we attempted to classify etiology by using ICD-10 codes I63.0–I63.9 and I64, there was a limitation in classifying stroke etiology by ICD-10 code due to the low prevalence of ischemic stroke. For example, cerebral vein thrombosis is one of the causes for ischemic stroke, but the number of diagnoses was low for analysis. The classification of patients according to each ward was inaccessible.

We are extremely sorry for not being able to meet the editor’s demands due to the limitations and anonymity of the data. However, in this paper, we used large-scale data using 10% of the Korean population and data using a 12-year-long follow-up. Our nationwide longitudinal cohort study provides information about the association between RA and ischemic stroke.

**Comment c)**

**c) Clear definition on each risk factor should be added (i.e. hypertension)**

**Author’s Response:**

We agree that it is crucial to define clearly each risk factor. Among the seropositive RA and control groups, ischemic stroke patients were those with ICD-10 codes I63 or I64, hospitalized for ischemic stroke, as confirmed by brain CT or MRI scans. Hypertension was defined as ICD-10-CM codes I10–I13 and I15 and a minimum prescription of one of anti-hypertensive drugs (thiazide, loop diuretics, aldosterone antagonist, alpha-/beta-blocker, calcium-channel blocker, angiotensin-converting enzyme inhibitor, angiotensin II receptor blocker). Additionally, diabetes mellitus was defined as ICD-10-CM code E11-E14 and the prescription of anti-diabetic drugs (sulfonylureas, metformin, meglitinides, thiazolidinediones, dipeptidyl peptidase-4 inhibitors, α-glucosidase inhibitors, and insulin). Dyslipidemia was defined as at least one claim per year for the prescription of anti-dyslipidemic medication under ICD10 code E78. We mentioned and provided a table of definitions of ischemic stroke and comorbidities as follows (Table 1).

**Table 1. Definitions of Ischemic stroke and comorbidities**

| **Diagnosis** | **ICD-10 codes** | **ICD-10 codes and definitions** |
| --- | --- | --- |
|  |  |  |
| Hypertension^a^ | I10-I13, I15 | At least one claim per year for the prescription of Anti-hypertensive medication (thiazide, loop diuretics, aldosterone antagonist, alpha-/beta-blocker, calcium-channel blocker, angiotensin-converting enzyme inhibitor, angiotensin II receptor blocker) under ICD-10 code I10-13 or I15. |
| Type 2 DM^b^ | E11-E14 | At least one claim per year for the prescription of  Anti-diabetic medication (sulfonylureas, metformin, meglitinides, thiazolidinediones, dipeptidyl peptidase-4 inhibitors, α-glucosidase inhibitors, and insulin) under ICD-10 code E11-14. |
| Dyslipidemia^b^ | E78 | At least one claim per year for the prescription of anti-dyslipidemic medication under ICD10 code E78 |
| Ischemic Stroke | I63, I64 | Hospitalized ≥ 1 day for ischemic stroke, as confirmed by brain CT or MRI scans |

^a^ ≥ 1 diagnosis during hospitalization, or ≥ 2 diagnoses at outpatient clinic, in the previous 1 year.

b ≥ 1 diagnosis during hospitalization or at outpatient clinic, in the previous 1 year.

**Page 5, line 120**

Among the seropositive RA and control group, ischemic stroke patients were those with ICD-10 codes I63 or I64; hospitalized for ischemic stroke, as confirmed by brain CT or MRI scans [22,28]; and hospitalization ≥1. In the seropositive RA group, ischemic stroke patients were limited to the case of receiving the ICD-10 codes I63 or I64 after diagnosed with the M5. Comorbidities such as diabetes mellitus (E11–E14), hypertension (I10–I13, I15), and dyslipidemia (E78) were defined by ICD-10 codes with additional information described previously [3-5] (Table 1).

**RESULTS**

**Comment a)**

a) **P values are missing, on each one of the tables (HR). When adding statistical analysis for association, you should provide this values.**

**Author’s Response:**

We appreciate your valuable comment. Thanks to your comment, we added P values on each table (HR). We have revised Tables 2 and 3.

**Table 2. Adjusted hazard ratio for ischemic stroke in the seropositive RA and control group**

| **Disease** | **Group** | | **N** | **Event** | **Duration** (days) | **Incidence rate** (1000 person-year) (%) |  | | |  | | | **Hazard Ratio**  (95% CI) | | | |
| --- | --- | --- | --- | --- | --- | --- | --- | --- | --- | --- | --- | --- | --- | --- | --- | --- |
|  |  |  |  |  |  |  | **Model 1** | **P values** | | | **Model 2** | | | **P**  **values** | |  |
| Stroke | | Control | 13,825 | 512 | 55273331 | 3.38 | 1 | | 0.039 | | | 1  1.40  (1.07–1.82) | | | 0.014 | |
|  | | RA | 2,765 | 64 | 5590704 | 4.18 | 1.32  (1.02–.73) | |  | | |  |  |  |  | |

Abbreviations: CI, confidence interval; RA, rheumatoid%d arthritis

Model 1: Adjusted for age, sex

Model 2: Adjusted for age, sex, income, diabetes, hypertension, dyslipidemia

**Table 3. Subgroup analyses between seropositive RA and control group**

| **Variables** | | **Seropositive RA** | | **Control** | | **Hazard Ratio**  (95% CI) |  |
| --- | --- | --- | --- | --- | --- | --- | --- |
|  |  | **N** | **Incidence**  **Rate**  (1000 person-  year) (%) | **N** | **Incidence**  **Rate**  (1000 person-  year) (%) |  | P-values |
| Sex | Male | 18 | 4.80 | 178 | 4.65 | 1.12 (0.682-1.835) | P<0.001 |
|  | Female | 46 | 4.07 | 334 | 2.95 | 1.44 (1.048-1.971) |  |
| Age | <65 | 38 | 2.81 | 333 | 2.49 | 1.30 (0.922-1.832) | P<0.001 |
|  | ≥65 | 26 | 14.33 | 179 | 1.02 | 1.37 (0.900-2.082) |  |
| Diabetes | N | 7 | 0.49 | 100 | 0.73 | 1.47 (1.106-1.952) | P<0.001 |
|  | Y | 57 | 59.60 | 412 | 28.14 | 0.98 (0.452-2.124) |  |
| Hypertension | N | 29 | 2.72 | 240 | 2.48 | 1.14 (0.769-1.687) | P<0.001 |
|  | Y | 35 | 7.54 | 272 | 4.99 | 1.66 (1.156-2.378) |  |
| Dyslipidemia | N | 55 | 4.27 | 403 | 3.24 | 1.43 (1.073-1.912) | P<0.008 |
|  | Y | 9 | 3.69 | 109 | 4.02 | 0.92 (0.462-1.833) |  |

Abbreviations: RA, rheumatoid arthritis; CI, confidence interval

**Comment b)**

**b) Kaplan Meier Curve should be corrected: Log-rank p value should be included, On the X axis, the authors should clarify if they are using person-year or years. Number at risk table should be included for each group, below X axis on each point of the analysis.**

**Author’s Response:**

We appreciate your comment. We have revised the Kaplan–Meier curve to include the log-rank p value. On the X-axis, we used days. We have referenced and corrected the figures to avoid confusion.

**Page 10, line 171**

The incidence rate of ischemic stroke was higher in the seropositive RA group (4.18) than in the control group (3.38) per 1000 person-year. The hazard ratios of ischemic stroke in the seropositive RA group were 1.32 (95% CI, 1.02–1.73) and 1.40 (95% CI, 1.07–1.82) according to models 1 and 2, respectively (Table 2). The cumulative incidence of ischemic stroke was significantly higher in the seropositive RA group than that in the control group (p =0.014, Fig 2).

**Fig 2. Comparison of the cumulative incidence of ischemic stroke in the seropositive RA and control groups**. The Kaplan-Meier curves with cumulative hazards of ischemic stroke were compared between seropositive RA and control groups (*p* =0.014)

**Comment c)**

**c) When including data from severity, type of stroke, and acute therapy I suggest to run again your analysis, to evaluate if the previous findings still remain in terms of covariates.**

**Author’s Response**:

We appreciate your valuable comment and understand your concern. Since deidentified data was provided, we could not obtain all of each individual’s clinical information from the NHIS-HEALS cohort. It was impossible to know the mortality and functional outcomes according to the severity of stroke cases. Unfortunately, for the analysis of stroke type and acute therapy, longer follow-up data were required to overcome the low incidence of ischemic stroke; however, further analysis was not possible for above mentioned reasons and the long waiting time of further approval to access the database.

**Comment d)**

**d) Finally, the previous changes should be considered in terms of the Discussion section, if significant changes are found.**

**Author’s Response**:

I really appreciate your valuable comment. Even though no significant changes were found, we added a discussion on the relationship between atrial fibrillation, one of the major cause of ischemic stroke, and RA.

**Page 12, line 223**

Atrial fibrillation is a crucial and remediable risk factor for stroke [6]. The incidence of atrial fibrillation is known to increase due to several more common factors in RA patients, such as smoking, ischemic heart disease, and heart failure [7-10]; Nonetheless, the relationship between atrial fibrillation and ischemic stroke is uncertain in RA patients [11]. However, research has shown that inflammatory reactions increase atrial fibrillation [12], and the increased risk of atrial fibrillation is expected to play an important role in the development of ischemic stroke in RA patients. To better understand the relevance of risk factors associated with ischemic stroke and RA, researchers should address the degree of involvement of risk factors.

**Reviewer #1:** In the introduction part you have to explain better the definition of rheumatoid arthritis in which it doesn’t describe the dysfunction produced by it.
It is important the mention the definition of seropositive rheumatoid arthritis and what kind of antibodies were used to classify the population.
In the study design and subjects would be better if you explain what kind of DMARD was prescribed (biological or synthetic)

**Author’s Response**:

We agree with your comment. The presence of anti-cyclic citrullinate peptide (CCP) is hallmark of rheumatoid arthritis (RA) along with rheumatoid factor (RF), and is part of the 2010 American College of Rheumatology (ACR)/European League Against Rheumatism (EULAR) classification criteria.[13]. Patients with positive for CCP antibody and/or RF may be referred to as “seropositive” and make up approximately two thirds of the RA population.

Rheumatoid factor (RF) and anti-cyclic citrulline peptide (CCP) antibody tests are often measured when diagnosing rheumatoid arthritis (RA), but may not be repeated, making it difficult to extract directly from electronic health record data. Considering these points, we used M5 (seropositive) diagnostic ICD-10 code. Approximately 90% of RA patients were coded consistently using either M05 (seropositive) or M06 (seronegative). Under ICD-10 codes, M05 and M06 diagnosis codes are reasonable to identify seropositive and seronegative RA with high sensitivity and positive predictive values if lab test results are not available [2].

We monitored cohort participants for the development of RA as determined by ICD-10 codes M05–M06 in combination with prescription of disease modifying antirheumatic drugs within 1 year before or after the time of diagnosis. This method has previously been validated with both sensitivity and specificity of greater than 80% [14].

Our study based on people who received conventional synthetic DMARD prescriptions such as hydroxychloroquine (70.20%), methotrexate (66.42%), sulfasalazine (35.23%), and leflunomide (23.24%). Biological DMARD medications were examined whether adalimumab (2.23%), etanercept (1.46%), infliximab, or rituximab were prescribed.

**Page 4, line 95**

Rheumatoid factor (RF) and anti-cyclic citrulline peptide (CCP) antibody tests are often measured to diagnose rheumatoid arthritis (RA); however, when we consider the tests may not be repeated, it is difficult to extract correctly from electronic health record data. Considering these points, we used M5 (seropositive) diagnostic ICD-10 code based on these factors [2].

**Page 5, line 102**

Our study based on people who received conventional synthetic DMARD prescriptions such as hydroxychloroquine, methotrexate, sulfasalazine, and leflunomide. Biological DMARD medications were examined whether adalimumab, etanercept, infliximab, or rituximab were prescribed.

**Reviewer #2**: This work approaches ischemic stroke in RA patients, a relevant complication considering the increased cardiovascular risk. It describes the incidence of ischemic stroke in Korean RA patients, obtaining data from a national database. Although it draws some interesting conclusions regarding stroke incidence and its associations, there are important aspects to be addressed.

**Major points**

**Comment 1)**
1) I consider the most concerning point is that there are contradictory statements regarding the main objective of the study (incidence of stroke in RA patients). In lines 136 and 137, the statement is: “The incidence of ischemic stroke was lower in the seropositive RA group (2.31%, 64 patients) than in the control group (3.70%, 512 patients)”; whereas in lines 142 and 143, it is: “The incidence of ischemic stroke was significantly higher in the seropositive RA group than in the control group (p =0.014, Fig 2).” Did the authors mean “cumulative incidence” in the second statement? If they were referring to person-years, they must clarify and include that information in the text, and not only in the figure.

**Author’s Response**:

We appreciate your comment. After your comment, we have corrected and revised the words to clarify meaning in the text, not only in the figure. The prevalence of ischemic stroke was lower in the seropositive RA group (2.31%, 64 patients) than that in the control group (3.70%, 512 patients). However, in the incidence rate using person-year, the incidence of ischemic stroke was higher in the seropositive RA group (4.18) than that in the control group (3.38) per 1000 person-year (Table 2). The cumulative incidence of ischemic stroke was significantly higher in the seropositive RA group than that in the control group (p =0.014, Fig 2).

**Page 10, line 171**

The incidence rate of ischemic stroke was higher in the seropositive RA group (4.18) than in the control group (3.38) per 1000 person-year. The hazard ratios of ischemic stroke in the seropositive RA group were 1.32 (95% CI, 1.02–1.73) and 1.40 (95% CI, 1.07–1.82) according to models 1 and 2, respectively (Table 2). The cumulative incidence of ischemic stroke was significantly higher in the seropositive RA group than in the control group (p =0.014, Fig 2).

**Fig 2. Comparison of the cumulative incidence of ischemic stroke in the seropositive RA and control groups**. The Kaplan–Meier curves with cumulative hazards of ischemic stroke were compared between seropositive RA and control groups (*p* =0.014)

**Comment 2)**
2) Authors should clarify why they included only patients with newly diagnosed RA, and not with prevalent RA. This could lead to bias and to a lower risk of ischemic stroke, since patients with a longer disease duration could have an even higher risk than those who were newly diagnosed in the established time period.

**Author’s Response**:

We appreciate your valuable comment and agree with your opinions. The NHIS-HEALS cohort covers approximately 10% of the Korean population from 2002 to 2003, aged between 40 and 79 and who had undergone national health screening. Among them, we studied patients with newly diagnosed RA. It was followed up for 12 years, until December of 2015. At the time of diagnosis, previously diagnosed RA patients had an increased prevalence of carotid plaque compared to controls [15, 16]. Nevertheless, if we include previously diagnosed RA patients, it is not possible to know the diagnosis time of RA. Therefore, it may be difficult to know the incidence rate and cumulative incidence of stroke with RA. In order to decrease the error due to a short period of time, the correlation between RA and ischemic stroke was analyzed with a sufficiently long follow-up of 12 years.

**Comment 3 :**

3. Authors should clarify whether the ischemic stroke was diagnosed after RA. Although that seems intuitive, it is not included in the Methods section.

**Author’s Response:**

I appreciate you for these helpful suggestions. We searched for patients diagnosed with ischemic stroke by 2015 after diagnosed with RA. We added this detail to the Materials and methods section.

**Page 5, line 119**

Among the seropositive RA and control group, ischemic stroke patients were those with ICD-10 codes I63 or I64; hospitalized for ischemic stroke, as confirmed by brain CT or MRI scans [22,28]; and hospitalization ≥1. In the seropositive RA group, ischemic stroke patients were limited to the case of receiving the ICD-10 codes I63 or I64 after diagnosed with the M5.

**Minor points:**
1. Line 85: “Accessed the databases number of this paper is NHIS-2019-2-194.” This statement does not make sense.

**Author’s Response:**

I appreciate you for these helpful suggestions. I deleted the above sentence in accordance with your comment.

2. Line 97: There is a ”27” which seems out of place.

**Author’s Response:**

We agree with your comment. This was deleted.

3. Lines 134 and 135: although the authors mention that there were significant differences in the prevalence of diabetes, hypertension, dyslipidemia and stroke, they do not clarify which were more prevalent in the RA patients and which in the control group. Even though that information can be found on Table 3, since there are differences in each condition, I consider it should be added to the text.

**Author’s Response:**

We appreciate your comment and agree with your opinions. We also think that it is better to comment about each condition in the text. We have added the comments as follows.

**Page 9, line 160**

Significant differences were found between the two groups in the prevalence of diabetes, hypertension, dyslipidemia, and ischemic stroke. Diabetes prevalence was higher in the control group (10.10%) than in the RA group (6.26%) (p <0.001). Additionally, hypertension prevalence was higher in the RA group (30.13%) than in the control group (26.44%) (p <0.001). Dyslipidemia prevalence was higher in the control group (17.84%) than in the RA group (15.55%) (p = 0.004). Ischemic stroke prevalence was higher in the control group (3.70%) than in the RA group (2.31%) (p <0.001) (Table 4).

4. Line 192: Is it atrial fibrosis or atrial fibrillation? Authors should also clarify why would that explain a higher risk of stroke in RA patients

**Author’s Response:**

We appreciate your valuable comment. The previously mentioned word was atrial fibrillation. We revised our review of atrial fibrillation in the discussion as follows.

**Page 12, line 223**

Atrial fibrillation is a crucial and remediable risk factor for stroke [6]. The incidence of atrial fibrillation is known to increase due to several more common factors in RA patients, such as smoking, ischemic heart disease, and heart failure [7-10]; nonetheless, the relationship between atrial fibrillation and ischemic stroke is uncertain in RA patients [11]. However, research has shown that inflammatory reactions increase atrial fibrillation [12], and the increased risk of atrial fibrillation is expected to play an important role in the development of ischemic stroke in RA patients. To better understand the relevance of risk factors associated with ischemic stroke and RA, researchers should address the degree of involvement of risk factors.

5. Line 203: What do you mean by “radiation grade”?

**Author’s Response:**

We appreciate your valuable comment .Thanks to your comment, we have revised the word to “radiographic findings” as follows.

**Page 13, line 239**

Furthermore, we were unable to provide information on RA clinical severity, such as disease activity score or radiographic findings.

6. Because of the nature of the study, we do not know the smoking status of the population. Since smoking is a risk factor for both RA and stroke, this should be discussed as a limitation.

**Author’s Response:**

I appreciate your valuable comment and I understand your concern. As your comment, we added comments that this study has inevitable limitation of not providing information on life-related factors such as smoking or alcohol consumption, physical activity, eating patterns, and obesity.

**Page 13, line 237**

There were several limitations to this study. Due to the basic limit of the NHIS database, information was lacking with regard to life-driven factors such as smoking or alcohol consumption, physical activity, eating patterns, and obesity.

7. The different prevalence of diabetes and dyslipidemia between both groups should be discussed.

**Author’s Response:**

I appreciate you for these helpful suggestions. After your comment, we added comments about each condition including hypertension and diabetes, as follows.

**Page 9, line 160**

Significant differences were found between the two groups in the prevalence of diabetes, hypertension, dyslipidemia, and ischemic stroke. Diabetes prevalence was higher in the control group (10.10%) than in the RA group (6.26%) (p <0.001). Additionally, hypertension prevalence was higher in the RA group (30.13%) than in the control group (26.44%) (p <0.001). Dyslipidemia prevalence was higher in the control group (17.84%) than in the RA group (15.55%) (p = 0.004). Ischemic stroke prevalence was higher in the control group (3.70%) than in the RA group (2.31%) (p <0.001) (Table 4).

**References**

1.	Seong SC, Kim YY, Park SK, Khang YH, Kim HC, Park JH, et al. Cohort profile: the National Health Insurance Service-National Health Screening Cohort (NHIS-HEALS) in Korea. BMJ Open. 2017;7(9):e016640. Epub 2017/09/28. doi: 10.1136/bmjopen-2017-016640. PubMed PMID: 28947447; PubMed Central PMCID: PMCPMC5623538.

2.	Curtis JR, Xie F, Zhou H, Salchert D, Yun H. Use of ICD-10 diagnosis codes to identify seropositive and seronegative rheumatoid arthritis when lab results are not available. Arthritis Research & Therapy. 2020;22(1):242. doi: 10.1186/s13075-020-02310-z.

3.	Lee SR, Choi EK, Han KD, Cha MJ, Oh S. Trends in the incidence and prevalence of atrial fibrillation and estimated thromboembolic risk using the CHA(2)DS(2)-VASc score in the entire Korean population. Int J Cardiol. 2017;236:226-31. Epub 2017/02/25. doi: 10.1016/j.ijcard.2017.02.039. PubMed PMID: 28233629.

4.	Lee SR, Choi EK, Han KD, Cha MJ, Oh S, Lip GYH. Temporal trends of antithrombotic therapy for stroke prevention in Korean patients with non-valvular atrial fibrillation in the era of non-vitamin K antagonist oral anticoagulants: A nationwide population-based study. PLoS One. 2017;12(12):e0189495. Epub 2017/12/21. doi: 10.1371/journal.pone.0189495. PubMed PMID: 29261716; PubMed Central PMCID: PMCPMC5738023 Consultant for Bayer/Janssen, BMS/Pfizer, Biotronik, Medtronic, Boehringer Ingelheim, Microlife and Daiichi-Sankyo. Speaker for Bayer, BMS/Pfizer, Medtronic, Boehringer Ingelheim, Microlife, Roche and Daiichi-Sankyo. No fees are received personally. This does not alter our adherence to PLOS ONE policies on sharing data and materials.

5.	Park CS, Choi EK, Han KD, Lee HJ, Rhee TM, Lee SR, et al. Association between adult height, myocardial infarction, heart failure, stroke and death: a Korean nationwide population-based study. Int J Epidemiol. 2018;47(1):289-98. Epub 2017/10/13. doi: 10.1093/ije/dyx175. PubMed PMID: 29025084.

6.	Camm AJ, Kirchhof P, Lip GY, Schotten U, Savelieva I, Ernst S, et al. Guidelines for the management of atrial fibrillation: the Task Force for the Management of Atrial Fibrillation of the European Society of Cardiology (ESC). Eur Heart J. 2010;31(19):2369-429. Epub 2010/08/31. doi: 10.1093/eurheartj/ehq278. PubMed PMID: 20802247.

7.	Goldstein LB, Bushnell CD, Adams RJ, Appel LJ, Braun LT, Chaturvedi S, et al. Guidelines for the primary prevention of stroke: a guideline for healthcare professionals from the American Heart Association/American Stroke Association. Stroke. 2011;42(2):517-84. Epub 2010/12/04. doi: 10.1161/STR.0b013e3181fcb238. PubMed PMID: 21127304.

8.	Boyer JF, Gourraud PA, Cantagrel A, Davignon JL, Constantin A. Traditional cardiovascular risk factors in rheumatoid arthritis: a meta-analysis. Joint Bone Spine. 2011;78(2):179-83. Epub 2010/09/21. doi: 10.1016/j.jbspin.2010.07.016. PubMed PMID: 20851020.

9.	Wolfe F, Michaud K. Heart failure in rheumatoid arthritis: rates, predictors, and the effect of anti-tumor necrosis factor therapy. Am J Med. 2004;116(5):305-11. Epub 2004/02/27. doi: 10.1016/j.amjmed.2003.09.039. PubMed PMID: 14984815.

10.	Nicola PJ, Crowson CS, Maradit-Kremers H, Ballman KV, Roger VL, Jacobsen SJ, et al. Contribution of congestive heart failure and ischemic heart disease to excess mortality in rheumatoid arthritis. Arthritis Rheum. 2006;54(1):60-7. Epub 2005/12/31. doi: 10.1002/art.21560. PubMed PMID: 16385496.

11.	Lindhardsen J, Ahlehoff O, Gislason GH, Madsen OR, Olesen JB, Svendsen JH, et al. Risk of atrial fibrillation and stroke in rheumatoid arthritis: Danish nationwide cohort study. Bmj. 2012;344:e1257. Epub 2012/03/10. doi: 10.1136/bmj.e1257. PubMed PMID: 22403267; PubMed Central PMCID: PMCPMC3297675 at www.icmje.org/coi_disclosure.pdf (available on request from the corresponding author) and declare: no support from any organisation for the submitted work; no financial relationships with any organisations that might have an interest in the submitted work in the previous three years; no other relationships or activities that could appear to have influenced the submitted work.

12.	Engelmann MD, Svendsen JH. Inflammation in the genesis and perpetuation of atrial fibrillation. Eur Heart J. 2005;26(20):2083-92. Epub 2005/06/25. doi: 10.1093/eurheartj/ehi350. PubMed PMID: 15975993.

13.	Aletaha D, Neogi T, Silman AJ, Funovits J, Felson DT, Bingham CO, 3rd, et al. 2010 Rheumatoid arthritis classification criteria: an American College of Rheumatology/European League Against Rheumatism collaborative initiative. Arthritis Rheum. 2010;62(9):2569-81. Epub 2010/09/28. doi: 10.1002/art.27584. PubMed PMID: 20872595.

14.	Singh JA, Holmgren AR, Noorbaloochi S. Accuracy of Veterans Administration databases for a diagnosis of rheumatoid arthritis. Arthritis Rheum. 2004;51(6):952-7. Epub 2004/12/14. doi: 10.1002/art.20827. PubMed PMID: 15593102.

15.	Hannawi S, Haluska B, Marwick TH, Thomas R. Atherosclerotic disease is increased in recent-onset rheumatoid arthritis: a critical role for inflammation. Arthritis Res Ther. 2007;9(6):R116. Epub 2007/11/08. doi: 10.1186/ar2323. PubMed PMID: 17986352; PubMed Central PMCID: PMCPMC2246234.

16.	van Sijl AM, Peters MJ, Knol DK, de Vet HC, Gonzalez-Gay MA, Smulders YM, et al. Carotid intima media thickness in rheumatoid arthritis as compared to control subjects: a meta-analysis. Semin Arthritis Rheum. 2011;40(5):389-97. Epub 2010/10/05. doi: 10.1016/j.semarthrit.2010.06.006. PubMed PMID: 20889191.
