# Supplementary material for: Association between ischemic stroke and seropositive rheumatoid arthritis in Korea: A nationwide longitudinal cohort study
Source: PLoS One. 2021 May 17;16(5):e0251851. doi: 10.1371/journal.pone.0251851 (PMC8128246; doi:10.1371/journal.pone.0251851)
Supplement: Attachment — Submitted filename: Response to Reviewers.docx [file pone.0251851.s002.docx]

**Title of the Manuscript**:

**Association between ischemic stroke and seropositive rheumatoid arthritis in Korea: A nationwide longitudinal cohort study**

**Manuscript Number: PONE-D-20-30296R2**

Reviewer #2: The authors addressed my previous comments correctly. However, there are still some aspects I consider should be corrected:

Q: There are several grammatical errors throughout the manuscript, they should be corrected.

**Author’s Response:**

**I appreciate your valuable comment and I understand your concern. Following your advice, we have reviewed and corrected grammatical errors throughout the manuscript. Our original manuscript was edited by a native speaker of English. However, after your comment, our revised manuscript was edited again by another native speaker of English who also studied biology. Please refer to the certificate attached herewith.**

**Abstract:**
Q: The following statement is not clear: “Patients with seropositive rheumatoid arthritis (RA) who were matched in age and sex.”
Authors should state that they studied both RA patients and controls, who were age- and sex-matched.

**Author’s Response:**

**I appreciate and agree with your comment. We have changed the sentence according to your comment as follows.**

**Page 2, line 27**

**The purpose of this nationwide longitudinal follow-up study was to investigate the risk of ischemic stroke nationwide in** [**patients with seropositive rheumatoid arthritis**](https://europepmc.org/article/med/9058647) **(RA) and controls who were age- and sex- matched.**

Q: I consider the abstract conclusion is not accurate. “Due to the high risk of ischemic stroke, close observation is especially necessary in female, hypertension, non-diabetes, and non-dyslipidemia RA subgroups”.
Whereas RA patients who were female, hypertense and without diabetes or hypertension had a higher risk of strike than their non-RA counterparts, it does not mean that they were the group with the highest stroke risk. It should be interpreted that these patients, even without the traditional risk factors for stroke (except for hypertension), had a higher risk which could be potentially attributed to RA.

**Author’s Response:**

**I appreciate your valuable comments and agree with you. Thanks to your comment, we could revise abstract conclusion. We've revised the abstract conclusion as follows.**

**Page 2, line 45**

**RA patients in female, hypertension, non-diabetes, and non-dyslipidemia subgroups, even without the traditional risk factors for stroke (except for hypertension), had a higher risk of ischemic stroke, which could be potentially attributed to RA.**

**Introduction**
Q: This sentence does not make sense: “Unlike debilitating joint effects, RA is reported to be primarily associated with a decrease in median survival rate”. Please rephrase.

**Author’s Response:**

**I appreciate and agree that the sentence should be rephrased. We modified the sentence as follows.**

**Page 3 line 56**

**RA is reported to be associated with a decrease in median survival rate that is primarily due to cardiovascular complications.**

Q: Line 84 and 85: Change “who” for “that” and delete the “]” sign.

**Author’s Response:**

**I appreciate your comment and agree with you. We modified the sentence as follows.**

**Page 4 line 84**

**The NHIS compiled the NHIS-HEALS database representing a cohort that participates in national health examinations.**

Q: Line 95: it should be “citrullinated”

**Author’s Response:**

**I appreciate your very much. Thanks to your comment, we corrected the sentence as follows.**

**Page 4 line 95**

**Rheumatoid factor (RF) and anti-cyclic citrullinated peptide (CCP) antibody tests are often measured to diagnose rheumatoid arthritis (RA);**

Q: Line 122: Is it hospitalization >1 day?

**Author’s Response:**

**I appreciate your comment. The hospitalization is for 1 day or more than 1 day. It should be “hospitalization ≥1”. We revised it as follows.**

**Page 4 line 95**

**Among the seropositive RA and control group, ischemic stroke patients were those with ICD-10 codes I63 or I64; hospitalized for ischemic stroke, as confirmed by brain CT or MRI scans [22,30]; and hospitalization ≥1.**

**Results**

Q: Are incidence rates in Table 3 correct? In people with RA over 65 years of age, it is 14.33 vs 1.02 in controls, with no significant difference. Also, it is mentioned that patients with RA without diabetes had a higher risk than controls, and the incidence rate shown in the Table is lower on those patients (0.49 vs 0.73)

**Author’s Response:**

**I appreciate your comment and understand your concerns. In table 3, subgroup analyses were performed using Cox proportional hazards regression models to estimate the effects of seropositive RA on the risk of each event. Confidence interval of hazard ratio was affected by follow-up period. In over 65 year old subgroup, the follow-up period was short. Even though incidence rate in RA patients were high, short follow-up period may affect confidence interval of hazard ratio. In non-diabetes subgroup, the incidence number of ischemic stroke was small (N=7) and most cases occurred within short period of follow-up. These relative differences of follow-up period affected the confidence interval of hazard ratio.**

Q: The p value for hypertension in Table 3 should be in patients with hypertension, not without

**Author’s Response:**

**I appreciate your comment and understand your concerns. The P-values of the Table showed the difference between each comparative subgroup. For example, P-value was compared between hypertensive and non-hypertensive subgroups. After your comment, we revised the arrangement of the P-value within Table 3 as follows.**

**Table 3. Subgroup analyses between the seropositive RA and control groups**

| **Variables** | | **Seropositive RA** | | **Control** | | **Hazard Ratio**  **(95% CI)** | **P-values**  **for difference** |
| --- | --- | --- | --- | --- | --- | --- | --- |
|  |  | **N** | **Incidence**  **Rate**  **(1000 person-**  **year)** | **N** | **Incidence**  **Rate**  **(1000 person-**  **year)** |  |  |
| **Sex** | Male | 18 | 4.80 | 178 | 4.65 | 1.12 (0.682-1.835) | P<0.001 |
|  | Female | 46 | 4.07 | 334 | 2.95 | 1.44 (1.048-1.971) |  |
| **Age** | <65 | 38 | 2.81 | 333 | 2.49 | 1.30 (0.922-1.832) | P<0.001 |
|  | ≥65 | 26 | 14.33 | 179 | 1.02 | 1.37 (0.900-2.082) |  |
| **Diabetes** | N | 7 | 0.49 | 100 | 0.73 | 1.47 (1.106-1.952) | P<0.001 |
|  | Y | 57 | 59.60 | 412 | 28.14 | 0.98 (0.452-2.124) |  |
| **Hypertension** | N | 29 | 2.72 | 240 | 2.48 | 1.14 (0.769-1.687) | P<0.001 |
|  | Y | 35 | 7.54 | 272 | 4.99 | 1.66 (1.156-2.378) |  |
| **Dyslipidemia** | N | 55 | 4.27 | 403 | 3.24 | 1.43 (1.073-1.912) | P=0.008 |
|  | Y | 9 | 3.69 | 109 | 4.02 | 0.92 (0.462-1.833) |  |

Abbreviations: RA, rheumatoid arthritis; CI, confidence interval

Q: I would suggest deleting this statement “The hazard ratio of ischemic stroke among male patients in the seropositive RA group was 1.12 (95% CI, 0.68–1.84)”, since it was not significant and is shown in the Table.

**Author’s Response:**

I appreciate your comment. After your comment, we deleted the sentence.

**Discussion**

Q: The discussion is somewhat difficult to follow; it lacks cohesion and fails to give potential explanations and/or show the relevance of some of the study findings.

**Author’s Response:**

**I appreciate your opinion. After your comment, we revised the discussion part. We added a perspective about our findings on diabetes and hyperlipidemia. We mentioned the merits of our results compared to existing papers. The explanation about the mechanism of myocardial infarction with rheumatic disease was deleted. We corrected grammatical errors as well.**

**Page 11 line 197**

**We analyzed the association between seropositive RA and ischemic stroke in Korea. Our nationwide longitudinal cohort study showed an increased risk of ischemic stroke in patients with seropositive RA. Furthermore, we conducted a subgroup analysis.**

**Solomon et al. [11] reported that the adjusted relative risk of myocardial infarction, not ischemic stroke, was higher in women with RA than that in women without RA. Similarly, Turesson et al. [15] reported that there was a trend toward an increased incidence of first episodes of ischemic stroke in RA cohort; however, the difference was not statistically significant. In a nationwide Swedish rheumatology cohort, the hazard ratio for ischemic stroke was 1.29 (95% CI, 1.18–1.41) in RA patients [10]. These findings are similar to ours; in our study, the age- and sex-adjusted (model 1) hazard ratio of ischemic stroke in the seropositive RA group was 1.32 (95% CI, 1.02–1.73). In model 2 (which adjusted for comorbidities in addition to age and sex), the incidence rate of ischemic stroke was 1.40 (95% CI, 1.07–1.82).**

**Several studies have found no association between ischemic stroke and RA [11,36,37]. These results suggest a lack of power due to an insufficient number of ischemic stroke events or a weaker association between RA and ischemic stroke. Our nationwide longitudinal cohort study helped to address some of the above uncertainties.**

**According to the subgroup analysis, in RA patients with hypertension, the hazard ratios of ischemic stroke risks was 1.66 (95% CI, 1.16-2.38). Our results suggest that the risk of ischemic stroke increases in hypertensive RA patients. Hypertension may have caused damaging effects in the blood vessels of these patients. Accelerated atherosclerosis caused by inflammation may have increased arterial stiffness [41], which may explain the evolution of ischemic stroke risks observed in our cohort.**

**Our results showed that the prevalence of diabetes and hyprelipidemia was lower in the RA group than in the control group. The prevalence of diabetes was lower in the RA group (6.26%) than in the control group (10.10%) (p <0.001). The prevalence of hyperlipidemia was lower in the RA group (15.55%) than in the control group (17.84%) (p = 0.004). The above-mentioned results are similar to those reported in other studies [41,48]. One study [41] reported that diabetes mellitus tended to be less common in the RA group than in controls, and that the RA patients (4.6−6.3 mmol/L) had significantly lower total serum cholesterol than that in controls (5.4−7.0 mmol/L) (P < 0.001). A previous study [48] supported our results by reporting a lesser prevalence of hyperlipidemia in the RA group. In some studies [49,50], diabetes showed a higher prevalence in the RA group than in the control group, but there was no significant difference in the prevalence of hyperlipidemia between the two groups. There results demonstrates controversy in the prevalence of diabetes and hyperlipidemia among patients with RA compared to controls. Therefore, further studies may be warranted.**

**Smoking is a well-established risk factor for RA [38]. Moreover, smoking and inflammatory activity have been demonstrated to increase fibrinogen levels, and this increase is a known risk factor for thromboembolic events such as ischemic stroke [39]. Similar to smoking, hypercoagulation and thrombosis caused by the inflammatory reaction in RA, which leads to embolization, are described as factors that increase the risk of ischemic stroke in RA.**

**Page 14 line 256**

**Nevertheless, to the best of our knowledge, this is the first study conducted in Korea that evaluates the association between ischemic stroke and seropositive RA in Korean patients. Our study is a nationwide, longitudinal study which included a large national data set based on health check-ups, with a follow-up that provided unified data to overcome the limitations of existing research results that used small amounts of data [41,48,49,50]. A large number of RA patients and their age-sex matched controls in a ratio of 1:5 also helped overcome the limitations of very low ischemic stroke incidence.**

Q: As I mentioned in my previous comments, the different prevalence of diabetes and dyslipidemia between both groups should be added to the discussion (not just mentioned in the Results section).

**Author’s Response:**

**I appreciate you for these helpful suggestions. After your comment, the different prevalence of diabetes and dyslipidemia between both groups were added to the discussion as follows.**

**Page 12, line 220**

**Our results showed that the prevalence of diabetes and hyprelipidemia was lower in the RA group than in the control group. The prevalence of diabetes was lower in the RA group (6.26%) than in the control group (10.10%) (p <0.001). The prevalence of hyperlipidemia was lower in the RA group (15.55%) than in the control group (17.84%) (p = 0.004). The above-mentioned results are similar to those reported in other studies [41,48]. One study [41] reported that diabetes mellitus tended to be less common in the RA group than in controls, and that the RA patients (4.6−6.3 mmol/L) had significantly lower total serum cholesterol than that in controls (5.4−7.0 mmol/L) (P < 0.001). A previous study [48] supported our results by reporting a lesser prevalence of hyperlipidemia in the RA group. In some studies [49,50], diabetes showed a higher prevalence in the RA group than in the control group, but there was no significant difference in the prevalence of hyperlipidemia between the two groups. There results demonstrates controversy in the prevalence of diabetes and hyperlipidemia among patients with RA compared to controls. Therefore, further studies may be warranted.**

Q: The paragraph in lines 232 to 236 is not clear. The differential outcomes of myocardial infarction and stroke and then the different mechanisms leading to both of them do not appear to be related.

**Author’s Response:**

**I appreciate your comment and agree with you. After your comment, we deleted those sentences.**
